# A Recent Progress of Steel Bar Corrosion Diagnostic Techniques in RC Structures

**DOI:** 10.3390/s19010034

**Published:** 2018-12-21

**Authors:** Dong Luo, Yuanyuan Li, Junnan Li, Kok-Sing Lim, Nurul Asha Mohd Nazal, Harith Ahmad

**Affiliations:** 1 School of Human Settlements and Civil Engineering, Xi’an Jiao Tong University, Xi’an 710048, China; luodong@xjtu.edu.cn (D.L.); lijunnan0725@stu.xjtu.edu.cn (J.L.); 2 Photonics Research Centre, University of Malaya, Kuala Lumpur 50603, Malaysia; kslim@um.edu.my (K.-S.L.); n.asha2704@yahoo.com (N.A.M.N.); Harith@um.edu.my (H.A.)

**Keywords:** corrosion of steel bar (CSB), corrosion rate, corrosion current density, physical methods, electrochemical methods

## Abstract

Corrosion of steel bar is one of key factors undermining reinforced concrete (RC) structures in a harsh environment. This paper attempts to review the non-destructive procedures from the aspect of the corrosion measurement techniques, especially their advantages and limitations. Systematical classification of diagnostic methods is carried out to determine any probable corrosion issues before the structures become severe, and helps choose the suitable method according to different construction features. Furthermore, the three electrochemical factors method is introduced to inspire researchers to combine various techniques to improve corrosion evaluation accuracy. The recommendations for future work are summarized, in conclusion.

## 1. Introduction

**Figure 1:**
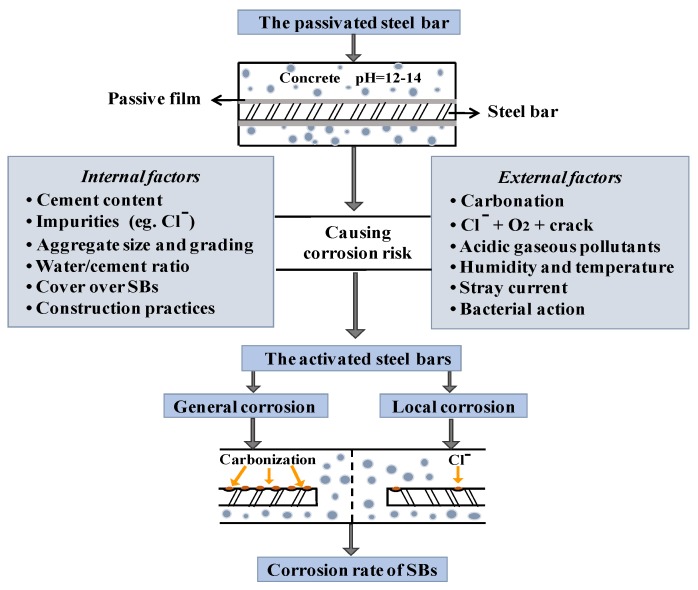
Corrosion process and mainly influencing factors of corrosion of steel bar (CSB).

Widespread premature deterioration of steel bar embedded in concrete (CSB), due to corrosion, is a major factor causing significant loss. Corrosion of CSB is caused by chemical or electrochemical reactions. Passivation film, as the first layer of defense, will be corroded by carbonation or chloride attack; then, the corrosion process propagates to the internal CSB. Once the deposited corrosion product reaches its threshold value, expansion stress leads to various types of damage, such as decreasing of the bonding strength between CSB and surrounding concrete, severely reducing the strength and remaining service life of RC structures [1]. Figure 1 shows the corrosion process and main influencing factors of CSB, providing significant information for the following discussion of influencing factors in corrosion diagnostic techniques.

Corrosion diagnostic technology is an essential tool to identify corrosion damage and validate the effectiveness of anti-corrosion measures. Initially, corrosion diagnostic technology was derived from corrosion tests in a laboratory, which always determined the corrosion status by weight loss of samples within a given time. In general, destructive testing (GWL) has achieved good results for the appraisement of the preservation status in RC structures; however, it is least preferred for dynamic corrosion diagnostic and assessment, due to its intrusive approach and induced collateral damage.

In order to avoid serious damage to structures, non-destructive testing (NDT) in engineering practice has attracted considerable attention, and mainly includes visual inspection, empirical analysis, physical testing and electrochemical testing. The first two methods mainly rely on certain detection tools and experience data to achieve corrosion identification. However, the last two kinds of techniques measure corrosion though changes in physical properties of CSB and electrochemical characteristics of corrosion reactions occurring in concrete.

The physical techniques have several limitations. Firstly, it is too late to perceive problems depending on the comprehensive method of visual inspection with the naked eye and physical techniques. Furthermore, the results cannot indicate corrosion information of the entire component due to the limited samplings. As corrosion is a result of electrochemical reaction, implying the transferring of electrical charge, electrochemical methods are of great concern, worldwide, for recognition of CSB corrosion and reflecting corrosion process in essence, because of simple-equipment, high-accuracy, and application in field tests. Considering the abovementioned numerous approaches to diagnosis of CSB corrosion, however, there is no general agreement on which assessment is the most accurate and appropriate one for corrosion extent measurement in RC structures. Hence, for practical engineering applications, the existing corrosion diagnostic technologies should be studied and compared in the following aspects for the purpose of corrosion detection and control. (1) Durability and reliability; (2) accuracy and reproducibility; (3) sufficient sensitivity and fast response speed; (4) comparatively simple operation and maintenance.

This paper introduces corrosion rate (CR) firstly, an important parameter for estimating corrosion status obtained by real measured weight loss and theoretical calculation values, respectively. Then, the available non-destructive techniques are reviewed from the aspect of CSB corrosion measurement, especially their advantages and limitations. Systematical classification of diagnostic methods is carried out to provide important information about any probable corrosion issues before structures become severe, and help in selecting the suitable method for different construction features and environments for engineers.

## 2. Evaluation Index of Steel Bar Corrosion

### 2.1. Gravimetric Weight Loss (GWL)

GWL is a fundamental destructive method to provide an accurate estimation for corrosion, based on the real measured weight loss before and after CSB corrosion. Meanwhile, GWL measurement in controlled laboratory setting has been deemed the gold standard test for various corroded steel structures, because of its high accuracy and ease-operation [2]. Much effort has been put into comprehensive experimental investigations, in particular to determine the relationships between gravimetrically measured CR and various electrochemical parameters, and, in turn, to verify accuracy and effectiveness of some electrochemical testing. 

According to ASTM G1-03 standard code [3], corroded CSB needs to be soaked about 25 min in the cleaning solution for rust removal on surface, which is a mixture of 1000 mL HCl with 50 g of SnCl_2_ and 20 g of Sb_2_O_3_. Then, the corrosion amount of CSB will be weighed. The CR is calculated as

(1) $$\nu \;=\;\frac{m_{i}{-m}_{f}}{St}\;=\;\frac{m_{i}{-m}_{f}}{\pi \mathrm{DL}t}$$

where ν is CR in ${g/(m}^{2}\cdot h)$; $m_{i}$ is the initial quality before corrosion in g; $m_{f}$ is the quality after corrosion in g; *S* is the corroded surface area of CSB in cm^2^; and *t* is the corroding time in hour; D and L are the diameter and length of steel bar in cm, respectively. The percentage of weight loss that determines the degree of corrosion ϱ is obtained by the following formula:

(2) $$\varrho \;=\frac{m_{i}{-m}_{f}}{m_{i}}\times 100$$

### 2.2. Corrosion Current Density (CCD)

CR obtained by GWL is calculated as average rate during the whole corrosion process; however, the instantaneous corrosion rate, CCD, experimented by electrochemical methods, is one key index to reflect corrosion status of CSB at a certain time. Based on Faraday’s law, corrosion current can be converted to loss of CSB [4]:

(3) $$\Delta m=\frac{I_{corr}t\omega_{m}}{VF}$$

where Δm is loss weight of CSB in g; *I_corr_* is corrosion current in A; $\omega_{m}$ is molar mass of Fe (55.847 in g/mol); *t* is corrosion time; *V* is the electrons transferred number (the value is 2); *F* is Faraday constant (96500 C/mol).

For uniform corrosion, surface of CSB can be regarded as anode area S, therefore, CCD will be calculated by $i_{corr}{=I}_{corr}/S$. The relationship between CR and CCD can be obtained in Equation (4). Furhtmore, the equivalent CCD, *i_corr_* (expressed in μA/cm^2^), is estimated as the subsequent Equation (5).

(4) $$\nu \;=\;\frac{\Delta m}{St}\;=\;\frac{\omega_{m}i_{corr}}{VF}$$

(5) $$\nu \;=\;{3.73\;\times \;10}^{-4}\;\times \;\frac{\omega_{m}i_{corr}}{VF}$$

## 3. Visual Inspection

Visual inspection is a qualitative assessment of CSB corrosion based on observation with naked eye, sometimes with tools of binoculars, magnifier, micrometer hammers, camera, etc., in a regular period of time according to the importance and age of structures. Recently, unmanned aerial vehicle (UAV) has successfully replaced the traditional detection tools, especially for special or long-span bridges. This technology combines field inspection in the first stage, and collected data analysis in second stage, to accurately identify corrosion status with more safety, efficiency, and at lower cost.

Usually, this method is a fundamental procedure to assess the preservation status of CSB as the first step for evaluation; however, the diagnosis merely based on the observable and exposed parts of structures is superficial. By the time that signs of damages, such as cracks and spalling, are detected, this indicates extensive corrosion in the hidden parts of the structures. Hence, it is desirable to initiate corrosion monitoring at the earliest stage during the construction phase, in a periodic manner, and using data logging.

## 4. Empirical Analysis

Empirical analysis aims at determining corrosion extent of CSB by considering, comprehensively, the external environment and internal factors in concrete mixture by certain deterministic and regression models. The in situ measured data used for models include common parameters of CSB diameter, protective layer thickness, concrete strength, content, and immersion depth of harmful ion, concrete temperature, exposure time, w/c ratio, cement content, etc., and special variables of loads. The accurate measurement of corrosion factors and the building of prediction model are the mostly key questions in this method, and the frequently used prediction models of CSB corrosion are as shown in Table 1.

As an intelligent diagnosis, ANN (artificial neural networks) is a powerful model-building tool using MATLAB function, due to its capabilities of stronger filtering, parallel processing, pattern recognition, and multi-input. Compared with the traditional models abovementioned, the ANN model-building process can learn from the experimental data, and then train itself to adapt to changing environments, which make ANN technology have bright application prospects with regard to automation and software. Since the 1980s, ANN has been successfully applied in many studies, such as identification of corrosion types (which include pitting corrosion [5], stress and crack corrosion [6]), determination of corrosion factors (Cl^−^) [7], and even analysis of spectral data from other corrosion diagnosis techniques [8]. Models based on ANN combined with other computational intelligence algorithms, such as imperialist competitive algorithm [9], BP (back propagation algorithm) [10], alternating conditional expectation algorithm [11], etc., offer more accuracy and flexibility in the detection of steel corrosion damage [12,13]. However, the application of ANN in CSB corrosion prediction is still rare. Hence, ANN technique has great room for development in many applications, such as corrosion failure analysis, image recognition of corrosion, etc., and to be combined with other powerful technologies, like expert system technology, database technology to increase its accuracy, universality of corrosion prediction.

Empirical analysis has been recognized as an efficient and economical method to provide qualitative evaluation for CSB loss by surveyor’s subjectivity. In general, it is considered as an auxiliary means of corrosion inspection, together with visual inspection or more powerful NDTs.

## 5. Physical Method

### 5.1. Electrical Resistance Probe (ERP)

**Figure 2:**
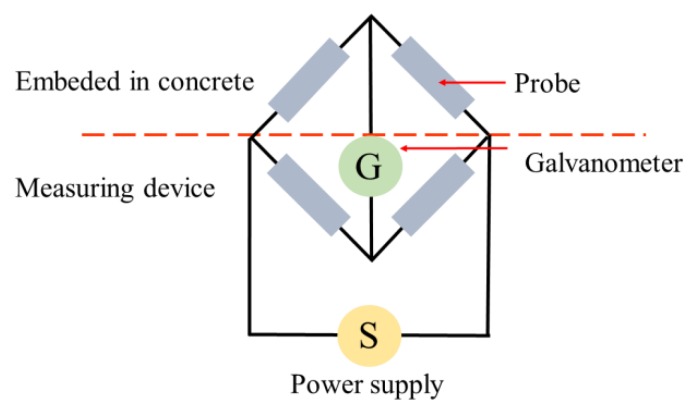
Measurement principle of electrical resistance probe (ERP).

In this technique, ERPs (of the same material–steel) are physically and electrically attached to CSB before concrete casting, and consist of four electrical resistances in a balanced bridge connection, as shown in Figure 2. Its resistance varies in inverse proportion to the cross-section area; therefore, changes in probe resistance can provide an indication to the thickness reduction by corrosion to achieve the test purpose. Furthermore, significant information about the corroded CSB of the cross-section reduction RCS, and the corroded layer thickness $T_{c}$, can be obtained [27]:

(6) $$\mathrm{RCS}\;=\;\frac{{\mathrm{CS}}_{0}\cdot \Delta R}{R_{0}}$$

(7) $$T_{c}\;=\;\frac{\mathrm{RCS}}{\mathrm{CF}}$$

where CS_0_ is the initial cross-section; *R*_0_ is the initial electrical resistance; ΔR is the change in electrical resistance of sensors during a certain time; CF is the initial circumference of sensors. 

Although, nowadays, applications of ERPs in concrete are limited, results have proven that they can provide an accurate and rapid estimation for general corrosion damage of CSB, due to their precision manufacturing process and circuitry design, and comparison with weightlessness experiments. ERPs are sensitive to physical parameters but not precisely responsive to changes in electrochemical parameters, which makes them suitable candidates for applications in all kinds of medium, regardless of the medium conductivity. For these reasons, they are primarily suitable for the validation of results from electrochemical sensors in corrosion measurements and monitoring CR and its change at all stages of structures in the long run [28].

On the other hand, ERPs can assess the availability of preventive methods, such as the corrosion inhibitor by Abed et al. [29]. Meanwhile, some novel ERPs (such as Qiao et al. [30]) were designed to monitor CR and even investigate the factors affecting measurement, in turn, to continuously evaluate the performance of ERPs by experimental results (such as Vieira et al. [31]).

### 5.2. Eddy Current Testing (ECT)

ECT is a widely used corrosion monitoring method, based on low frequency electromagnetic fields for inspecting the difference between surface of measuring devices and metal corrosion surface at high speeds without any physical contact between sensor and structure. CSB reaches magnetic saturation with the excitation of eddy current from electromagnetic devices, which are usually placed on concrete surfaces [32]. The cross-sectional reduction of CSB due to corrosion will cause abnormal phenomenon in magnetic field; therefore, corrosion information will be obtained with analysis of these variations. Usually, an eddy current system includes a single generator for energizing test coil sensor at optimum parameters of voltage and frequency, an oscilloscope to inspect signal quality, a digital desk-multimeter which measures voltage at the capacitive array, and Labview platform to automate measurement and record results, as shown in Ref. [33]. 

ECT is an effectual approach to identify loss of CSB with high speed, high accuracy, and in a quantitative way in harsh working environments where other methods are not suitable. Together with electrochemical detection, it can better diagnose the failure condition of concrete structures caused by CSB corrosion and even evaluate remaining service life, so it has great application prospects. Naasson et al. [33] and Minesawa et al. [34] has made great use of ECT on measurements for processes and position of corrosion in CSB, and Bailey et al. [35] studied wall loss defects of pipe with ECT.

Recently, many researchers have made efforts to develop novel electromagnetic sensors based on ECT technology, such as Alcantara et al. [36], combining ANN (artificial neural networks) analysis for locating and identifying CSB, and Rubinacci et al. [37], based on a non-iterative imaging method for the quantitative inspection of CSB corrosion. In addition, novel combination methods derived from conventional ECT were developed for application to direct corrosion detection and indirect characterization of certain electrochemical and physical properties of corroded CSB area (such as Suh et al. [38]; Yunze et al. [39]).

### 5.3. Acoustic Emission (AE)

**Figure 3:**
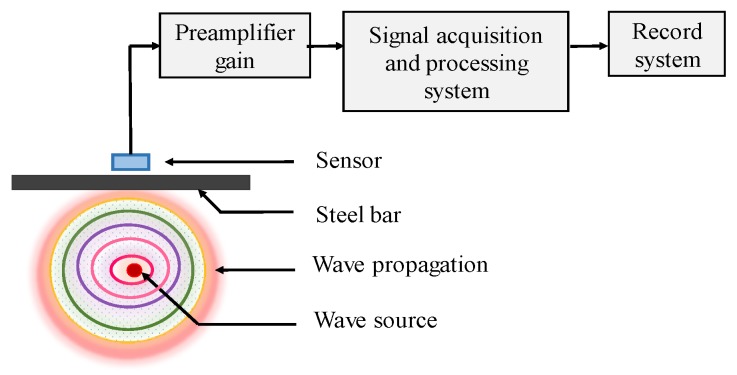
The principle of acoustic emission (AE).

AE has been extensively used in many areas, such as structure health monitoring (SHM) in bridge structures [40], and damage mechanism of plain concretes [41,42]. As the physical phenomenon, it can generate transient elastic waves rapidly though the energy release within structure. During corrosion process of CSB, the growing volume of corrosion products will induce stress to surrounding concrete, and eventually leads to crack or other forms of damage in concrete, thus generating sound waves. Therefore, AE sensors could determine location and intensity of the emission source (CSB corrosion expansion). The mechanical vibration will be converted into corresponding electrical signal; then, the signal is amplified by preamplifier, processed and recorded by a signal acquisition system, as shown in Figure 3.

Due to high sensitivity of AE, the dynamic process of corrosion can be studied well, and the damage of RC members evoked by CSB corrosion is expressed in the form of AE signal. Through online monitoring of accelerated corrosion test, Yu et al. [43] found that the overflow process of CSB corrosion produced obvious AE signals in concrete. Their analysis of AE signals shows that the average frequency is 20–30 kHz during failure of passivation film on CSB surface at a slight corrosion stage, while it is in the range of 30–50 kHz at the stage of concrete cracking caused by corrosion. To choose the AE sensors and set the parameters, Benedetti et al. [44] and Loreto et al. [45] have made good examples and provided relevant information.

AE signal is a powerful tool to provide assessment for damage condition of concrete, such as the location and, possibly, damage degree. Nonetheless, this method is susceptible to interference from other waves, and has difficulty in providing the quantitative relation between the corrosion level and AE signal. In recent years, many researches, such as Mangual et al. [46] and Elbatanouny et al. [47], made much efforts to quantify corrosion extent by AE. Calabres et al. [48] tried to denoise the environmental information for the purpose of long-time detection using AE by proposed algorithms for clustering and separation. However, these experiment results have no general agreement about corrosion in CSB and AE signals. 

In addition, AE technology is widely used in many aspects: (1) Interpretation of corrosion mechanisms (Qin et al. [49] and Kawasaki et al. [50]); (2) Identification of different sources of damage (Yoon et al. [51] and Wu et al. [52]), and damage evolution assessment in structural elements (Carpinteri et al. [53]); (3) Corrosion location in early stages and classification of different crack types (Elfergani et al. [54] and Ohtsu et al. [55]); (4) Lifetime estimation for the corroded beam (Zaki et al. [56]). Although the AE method is promising for CSB corrosion, some existing questions are worthy of further study, such as the removal of interference noise, AE dataset building (that is essential to definition of data mining-based filters in SHM), and application to on-site measurements.

### 5.4. Radiography

Radiography method is part of the reliable NDT techniques applied to produce images of CSB to obtain information about quality and defects within concrete. The basic principle is to convert the photons from radiation generator into visible light using a fluometallic converter. Two types of invisible electromagnetic radiations, namely, X-rays [57,58] and γ-rays [59], can penetrate and propagate through RC structures in straight paths without any significant diffraction. Irradiance of ray beams attenuate while propagating via the corroded material, thus producing the straightforward image guidance for CSB corrosion. In Ref. [60], the changes in diameter and presence of localized corrosion of CSB were successfully detected though radiography. 

Research by Dong et al. [61] has proved good correlation between the mass loss of CSB using X-ray, and that calculated by Faraday’s law, compared to GWL results. Usually, radiography technique is used as an auxiliary tool for study of CSB corrosion mechanisms to provide a visual assessment for hidden structures and guidance for maintenance and repair (such as Jensen et al. [62] and Meighen et al. [63]). In addition, this method provides a new angle to analysis of material composition (such as Šavija et al. [64]) and relative amounts of corrosion products (such as Ingham et al. [65]; Michel et al. [66]). 

However, it is restricted in concrete detecting, due to the inevitable shortcomings of high operational costs, low processing speed, bulky and costly equipment, strict safety protocol, precision interference, damage to buildings, limited dynamic range, and penetration depth. Moreover, Duffó et al. [60] found the detection limit of 90 μm in experiments of the present radiography work. For these reasons, radiography is not applicable to inspection of large structures, such as large beams, bridge piers, and dams.

### 5.5. Infrared Thermograph (IT)

In IT technology, photoelectric technology is applied to detect the specific infrared band signals of thermal radiation of object. It can convert the signals into images and graphics for visual resolution of humans. The principle is that CSB corrosion will cause changes in structures and composition of corroded parts, which will lead to different infrared radiation emitted by CSB. Continuous temperature distribution shows that there is no defect inside object, whereas the temperature gradient will appear on the surface of an object owing to defects inside its components. Therefore, IT can complete location identification and extent assessment of CSB corrosion intuitively, according to the temperature distribution on concrete surface. 

**Figure 4:**
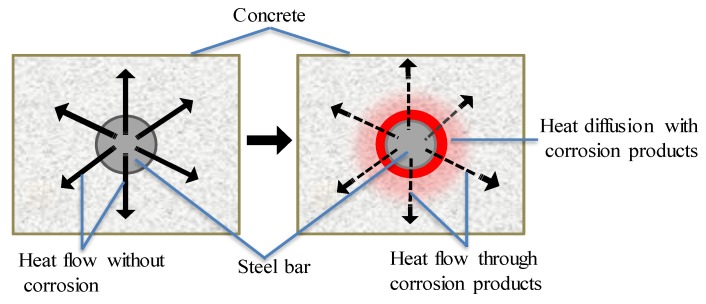
Effect of low heat corrosion products conductivity of steel corrosion products.

When an electromagnetic induction device is applied to heat CSB, there is smaller diffusion rate of heat from CSB to surrounding concrete, due to lower thermal conductivity of corrosion products in Figure 4. According to the different heat diffusion properties of CSB under various corrosion conditions, CSB subjected to induction heating can provide vital information about corrosion. The detection is easier with larger rebar diameter and smaller cover depths, because of faster heat diffusion and higher temperature increase on concrete surface. Kobayashi [67] obtained the infrared thermography images of temperature distributions on concrete surface under different corrosion loss using IT principle.

IT system is an automatic and real-time detection technology which allows inspection of large areas in a relatively short time. Meanwhile, it is an alternative method to acquire corrosion information from chloride content, which is a significant influencing element in RC structures, as the diffusion coefficient of chloride ion correlates well with heat dissipation characteristics of concrete [68,69]. Whereas, one drawback is that detectors of IT are susceptible to undesired radiation from the surrounding environment, while receiving infrared radiation from concrete. Therefore, there are significant challenges of the high requirement for environmental control and low reliability.

Numerous studies were carried out to assess applicability of IT technology and analyze corrosion defects existing in different structures, such as steel-concrete composite structures by Showunmi et al. [70], steel-concrete composite shear walls by Matovu et al. [71], concrete-filled steel tube by Xu et al. [72], oil conduits by Laaidi et al. [73], and organic coating layers by Jönsson et al. [74]. Furthermore, many scholars proposed novel NDT methods based on induction heating and IT camera in corrosion measurement for CSB, such as an integrated system combining electromagnetic heat induction and IR by Baek et al. [75] and Kwon et al. [76].

### 5.6. Fiber Optical Corrosion Sensors (FOCS)

Over the last two decades, FOCS have attracted substantial attention in SHM because of many intrinsic merits of high precision, being lightweight, flexible structure, corrosion resistance, multiple detection, immunity to electromagnetic interference, etc., compared to electronics-based sensors [77,78,79]. The principle of FOCS is based on use of photon transmission along the length direction in glass or organic fiber. The properties of light in optical fiber, including wavelength, energy flow density, frequency, polarization state, and phase, vary with changes in strain, temperature, and interface of fiber. In recent years, scholars combined physical changes and fiber sensing technology in the corrosion process of CSB, with repetitive exploration and continuous innovation, in order to realize the extensive applications of FOCS in the field of corrosion monitoring. 

Currently, the key FOCS methods include fiber Bragg grating, Brillouin optical time domain reflectometer/analysis, and long period fiber grating. In addition, some FOCS technologies have been applied extensively in experimental researches, such as optical time-domain reflectometer (OTDR) [80,81], Fabry–Perot interferometer [82,83], quasi-distributed sensors [84], Fe(C)-coated optical fiber sensors [85], etc. Nevertheless, there are existing problems for the wide application of all FOCS methods in practical engineering: (1) Quantitative relationship between changes in physical property and corrosion extent; (2) To avoid chemical attack, mechanical impact, or man-made interference, research on the packaging and embedding of sensors is particularly important; (3) Due to the limitation in the service life of optical fiber sensors, especially for glass fiber, the updating and replacement of techniques requires further research.

#### 5.6.1. Fiber Bragg Grating (FBG) Based on Strain Sensors

The 3–4 times volume variation compared to the initial steel, due to corrosion products, would cause tensile strain in optical fibers. After that, FBG sensors, which are usually fixed on CSB surface, can achieve the purpose of monitoring corrosion procedure and obtaining the corrosion intensity, based on changes in reflective index (RI) and grating period to shift their spectral response. Chen et al. [86] demonstrate good use of FBG principles. 

In an RC inspection context, FBG sensors provided a good indication of the reference corrosion rate for application in civil infrastructures, such as pre-stressed structures [87] and marine concrete structure [88]. Furthermore, some novel sensors were designed to show an example of great use of FBG. Gao et al. [89] proposed a novel sensor composed of one FBG sensing element and twin steel rebar elements, in order to investigate the relationship between reflected wavelength change from grating and weight loss rate by GWL. An FBG sensor, which is wrapped on steel bar with fiber-reinforced plastics, has been developed and validated by Zheng et al. [90] according to the principle of volume and diameter enlargement of CSB after becoming corroded. Recently, some research on Fe(C)-coated FBG technique by different preparation methods have been proposed, such as magnetron sputtering by Zhang et al. [91] and electroplating by Hu et al. [92]. Their research results proved that these sensors have great potential for corrosion alarm monitoring.

#### 5.6.2. Long Period Fiber Grating (LPFG) Based on RI Sensors

The gratings of LPFG sensor inscribed on fiber core produce a periodic refractive index modulation, about 100–1000 μm along the length of the fiber, to promote the light coupling from propagating core mode to co-propagating cladding modes at discrete wavelengths, which results in a series of attenuation bands in the transmission spectrum. Though considering the phase matching condition [93], the resonant wavelength changes with RI in a corrosive environment surrounding the gratings, and then corrosion information will be obtained according to the relationship between resonant wavelength changes and CR.

James S W et al. [93] has presented well the principles and fabrication of LPFG, and their results offered the bright prospect for designing comprehensive optical fiber sensors capable of simultaneous and independent monitoring for multiple measurands, due to their differing sensitivities to various measurands (such as RI, temperature, bending, strain, etc.). In application of LPFG, sensors coated with metal films play a very important role. Chen et al. [94,95] studied the LPFG corrosion sensor with Fe–C alloy film regarding its corrosion mechanism, sensing range, and even service life, and successfully applied sensors in corrosion monitoring of CSB based on changes in resonant wavelength as a function of corrosion-induced mass loss of steel bar. Preliminary results by Coelho et al. [96] show that Fe-coated LPFG has great potential to realize early-warning corrosion under a severely corroded conditions of coastal and offshore projects. A recent work by Huang et al. [97] proposed an LPFG sensor coated with a thin layer of polyurethane and nano iron/silica particles to monitor the corrosion process of deformed steel bar, which shows the wide application in LPFG sensors using coating technology. The better durability and wide application in different RC structures, by using the LPFG sensors, needs to be further investigated.

#### 5.6.3. Brillouin Optical Time Domain Reflectometer/Analysis (BOTDR/A)

**Figure 5:**
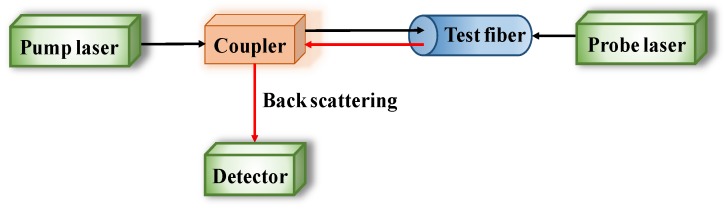
Configuration of Brillouin optical time domain reflectometer/analysis (BOTDR/A) analysis system.

Distributed optical fiber sensors, named BOTDR/A, are wound around CSB to reflect the corrosion process though corrosion expansion, based on the propagation of incident pulse train and Brillouin backscattering when light travels through the fiber in Figure 5. Sun [98] has presented, very well, the basic principles of BOTDR/A.

The unique ability of BOTDR/A technique is to continuously monitor the strain distribution and temperature, and it has successful and extensive applications in the bright corrosion field of CSB in RC structures [99]. Moreover, it inherits the sufficient monitoring range for CSB corrosion from the corrosion initiation until larger cracks occur in concrete, making it fully meet the requirements of practical engineering. Based on the distributed monitoring ability, BOTDR/A sensors play a significant role in the large-scale or long-span concrete component involved in bridge, dam, tunnel, and even ultrahigh-rise buildings, etc. In an article by Zhao et al. [100], the coil winding of optics fiber was proposed to directly measure strain change due to corrosion expansion, based on the distributed sensing ability of BOTDR/A. Mao et al. [101] introduced a novel monitoring method combining BOTDR/A for monitoring concrete expansion and FBG for identifying cracking, which realized synchronized monitoring of corrosion and cracks. 

However, this technique has some defects in application: (1) Its measurement precision is limited by the spatial resolution, which is defined as the smallest segment capable of discerning the average strain or temperature along optical fiber. Wei et al. [102] investigated the deformation resolving capability of BOTDR/A sensor using a low-coherent fiber-optic strain sensor (LCFS). (2) Although the simple packaging structure is conducive to practice application, its embedded installation technology in concrete is limited. To avoid major obstacles, the embedding technique using air-blowing and vacuum grouting provides an opportunity of placing long-distance FOCS into concrete without damage [103]. (3) Since the sensing section is in direct contacted with CSB surface, the sensors are easily destroyed, due to the appearance of large cracks in concrete. (4) Larger temperature change has a greater impact on its measurement results, hence, temperature compensation should be improved. 

## 6. Electrochemical Method

### 6.1. Half-Cell Potential Measurements (HCP)

Metal corrosion is the tendency of metal atoms to release electrons and form ions. This happens when metals are in contact with electrolyte, producing the potential that drives the corrosion process. In an RC system, concrete acts as an electrolyte, and corroded CSB develops a potential [104]. As the potential decreases with steel activation process, the potential difference between non-corroded area (cathode) and corroded (anode) area will be detected, forming a micro corrosion battery. Hence, the corrosion state of CSB can be identified using this principle.

**Figure 6:**
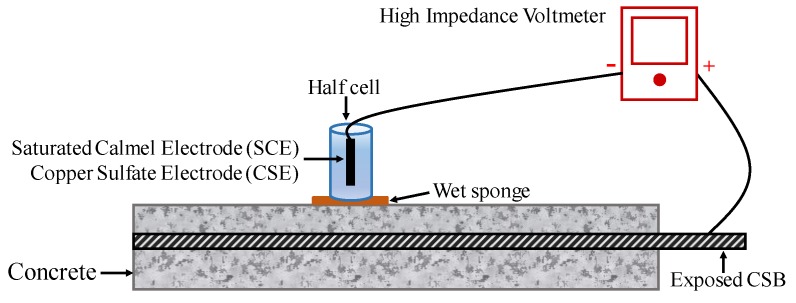
The setup of half-cell potential (HCP) measurements.

To conduct an HCP survey on RC structures with suspected corrosion is now a common practice, particularly due to chloride contamination. The potential of CSB might vary by corrosion, while that of the standard reference electrode is stable. Therefore, a change in potential can present a different corrosion status of CSB. Figure 6 shows the configuration of a half-cell system, which comprises of a metal rod soaked in a solution of the same metal anion (such as Ag/AgCl and Cu/CuSO_4_). A rod is electrically connected to CSB via a voltmeter. Sponge and surrounding concrete between half-cell and CSB are wet to ensure good electrical connection [105]. 

To evaluation corrosion, multiple point measurements and average data should be taken on the specimen surface to obtain a more precise result. Based on the extensive data collected in experimental structures, anode and cathode areas of CSB can be determined, thereby determining the corrosion position. For the standard reference electrode of SCE, a negative value less than 125 represents low risk corrosion; however, the negative value greater than 426 represents the severe corrosion condition. Hence, a more negative value of measured HCP indicates a higher chance of corrosion, and the probability of CSB corrosion can be found in ASTM C876 [106].

Potential mapping is a standardized open circuit potential for corrosion status assessment of CSB. In remediation and repair for RC, the position and affected area of corrosion should be identified over the entire structure. According to measured HCP values, establishing a potential map of structure surface is to diagnose the probability of corrosion at different points on the concrete surface. Results indicate that high negative potentials and dense equipotential lines represent the portions of structures’ likelihood of high corrosion activity.

Usually, HCP is applied to study corrosion mechanisms with other electrochemical methods, as it makes an evaluation of possibility and position of corrosion, and no quantitative assessment of CR [107]. There are numerous reports suggesting a reliable relationship between potential and CR, but they were mostly conducted in laboratory with well controlled conditions. It is highly probable that there could be large variations/error using the HCP method in such conditions: (1) The environmental condition of contamination with chloride, wetting concrete, and macrocell formation will result in a decrease in the potential over anode [108]. (2) There is the tough task of solid connection between CSB and densely reinforced members, like bridge decks [109]. (3) Many parameters, including composition and thickness of concrete cover, might influence the time required to establish an equilibrium condition between concrete and electrodes [110]. (4) CSB corrosion caused by leaching cannot be detected using HCP [111]. (5) Distance between CSB and the corrosion monitoring system deserves consideration. The experimental results by Jin et al. [112] show that the corrosion monitoring system should be located as close as possible to the working electrode to improve the measurement accuracy.

### 6.2. Concrete Resistivity Measurement

Corrosion, as an electrochemical process, involves concrete in the form of ions in the reaction area of two electrodes. After passivation of CSB, the corrosion speed depends on the supply of oxygen to the cathode reaction, and concrete resistivity, which determines the transfer velocity of ion between cathode and anode. Concrete resistivity is strongly related with the concrete properties, such as specimen geometry, microstructure, concrete pore saturation, and exposure conditions. Meanwhile, it can be improved by following methods: (1) Decrease of moisture content [113], chloride ion [114], and temperature; (2) Increase in time of hydration; (3) Carbonation process [115]; (4) Inclusion of SCMs, fly ash [116], and furnace dust [117]. Essentially, large-pore concrete filled with highly connective water is an indication of a high resistivity, and vice versa.

**Figure 7:**
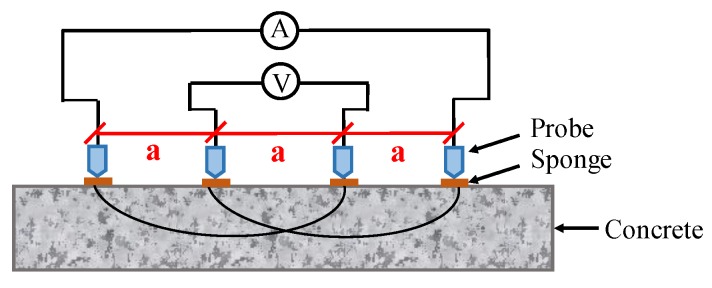
The schematic diagram of four-electrodes measurement for concrete resistivity.

Concrete resistivity can be measured using two different techniques, namely DC and AC techniques. Both surface and embedded probes are required in these measurements. For the DC technique, the measurement is achieved by performing a constant voltage difference on two embedded electrodes, and then detecting the induced electric current to determine resistance. For the AC technique, there are two approaches, namely two-pin method and four-pin method [118]. The four-point Wenner probe technique is a suitable candidate for long-term monitoring system in CSB corrosion, because it is a non-intrusive measurement that does not require a physical accessibility with the embedded rebar in the concrete. AC current is injected into the circuit through outer electrodes, and the potential between inner electrodes is measured as illustrated in Figure 7. Note that commercial Wenner probes mostly work at frequencies higher than 10 Hz, in order to reduce times and minimize noise during measurement [119].

The resistivity can be calculated according to

(8) $$\rho \;=\;2\pi \alpha \frac{\Delta V}{I}$$

where α is distance of inner electrodes, *I* is the impressed current on outer electrodes, and *V* is the measured potential. As a preliminary method, there is no definite equation or characterization curve between concrete resistivity and corrosion polarization current represents CSB corrosion; therefore, it can qualitatively determine corrosion process but cannot quantitatively evaluate the actual CR. For the four-pin method, the concrete resistivity more than 20 indicates the low corrosion rate; however, the value less than 5 indicates a very high corrosion rate. The corrosion rates with concrete resistivity measurement can be referred to in Ref. [118].

Concrete resistivity can serve as a supplementary measurement with other techniques for locating the problem areas or validating concrete quality [120]. However, it is susceptible to environmental perturbation. The measurement of concrete resistivity for various shapes and dimension are quite consistent and repeatable, provided that the electrodes used are well attached and installed on concrete, and the spacings between them are properly made. Salehi et al. [121] found that the appropriate position or orientation of probes can improve the accuracy by 30%. 

The analytical modeling methods for concrete resistivity have been studied in recent research. Yu et al. [122] proposed a probabilistic prediction model to calculate corrosion risk level of CSB based on concrete resistivity, in order to avoid the problem of wrong judgement in assessment by traditional deterministic methods. Huang et al. [123] developed a macrocell corrosion model to study the effect of concrete resistivity on CR, based on the Butler–Volmer equation and electrochemical principles. Presuelmoreno [124] realized the effective application of FEM in assessment of factors influencing apparent concrete resistivity in Wenner probe measurement. Lim et al. [125] built the resistivity estimation model (REM) to achieve quantitative evaluation of the geometric effect.

### 6.3. Linear Polarization Resistance (LPR) Measurement

LPR measurement, developed in 1957 by Stem and Geary, is a powerful tool to assess quantitatively galvanic and general corrosion. The theory of linear polarization is also recognized as polarization resistance (*R_p_*), where HCP of a piece of corrosion CSB is correlated to an external applied current, and CR is proportional to the applied current divided by change in potential. 

**Figure 8:**
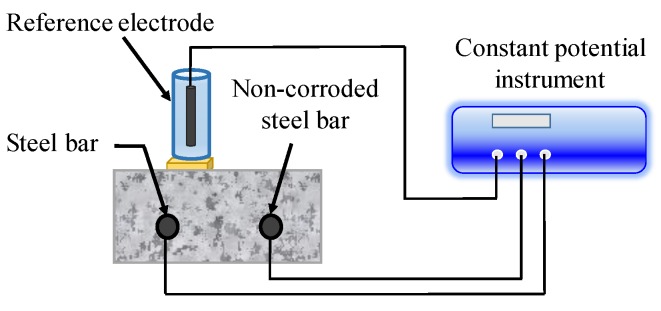
Linear polarization measurement system.

In LPR method, a small amount of equilibrium potential (10–30 mV) is applied between the reference electrode on concrete surface and CSB, as illustrated in Figure 8. The response current in a certain time is monitored, and the ratio of current to voltage amplitude is defined as the polarization resistance, *R_p_*. Besides, there are several methods for *R_p_* measurement, such as constant current method, dynamic potential method, and dynamic current method. Recently, a novel device of LPR, designed by Castillo et al. [126] for remote measurements of CSB corrosion, has a lower percentage of error compared to the equipment designed for commercial use.

**Figure 9:**
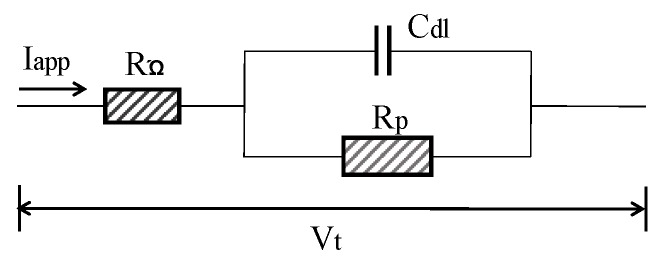
Randles model circuit.

In general, concrete system can be electrically characterized using Randles model circuit as depicted in Figure 9. *C_dl_* is the double layer capacitance of RC interface, *R_Ω_* is the concrete resistance between the reference electrode and CSB. The estimation of corrosion current is based on the Stern–Geary formula:

(9) $$B\;=\;\frac{\beta_{a}\cdot {\;\beta }_{c}}{{2.303(\beta }_{a}{+\;\beta }_{c})}$$

(10) $$i_{corr}\left({\mu A/\mathrm{cm}}^{2}\right)\;=\;\frac{B}{R_{p}}\;=\;\frac{\beta_{a}\cdot {\;\beta }_{c}}{{2.303(\beta }_{a}{+\;\beta }_{c}{)R}_{p}}$$

where $\beta_{a}$ $\beta_{c}$ are the Tafel constants of anode and cathode respectively, which can be determined by using Tafel diagram. *B* is the Stern–Geary constant, and its value is normally assumed to be 25 mV and 50 mV for active and passivity corrosion, respectively. From Faraday’s law, the corrosion current can be converted to the loss of CSB using Equation (3).

Corrosion is a fluctuating dynamic behavior, and interpretation of CR measurements should focus on the order of magnitude rather than the precise value obtained [127]. According to laboratory and field measured data, a corrosion current density *i*_corr_ between 10 µA/cm^2^ and 100 µA/cm^2^ indicates a very high corrosion rate. Typical corrosion rates for CSB and their corresponding corrosion penetration are shown in Ref. [128].

**Figure 10:**
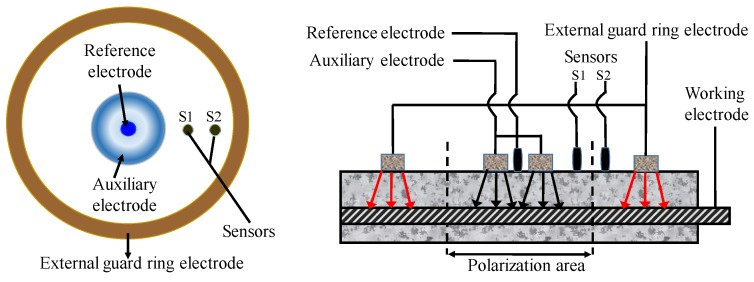
The portable ring measuring device.

In the application of this technology, several uncertain factors have to be taken into consideration, such as the measuring time, area of CSB, and compensation of insulation resistance (IR) drop. Concrete has great influence on determination of RP due to its high resistance. Hence, some measures must be taken to reduce IR drop. In recent years, IR compensation technology has become mature for small instruments though the automatic resistance compensation function [129]. However, at present, the polarization area is still a topic, due to the non-uniform polarization interval of CSB. The guard ring technology proposed by Feliu et al. [130] is the most advantageous solution to restrict polarization region of CSB to a known region by adding auxiliary electrodes in Figure 10. The polarization area of measured CSB is defined by maintaining the potential difference for two sensors S1 and S2 before and after disturbance, which is in the middle of the position of S1 and S2.

Results show that overcompensation or undercompensation easily occur when the electrode of the guard ring is used to compensate current, causing an inaccurate description of CSB corrosion. Dong et al. [131] proposed the principle of intelligent compensation and defined a compensation coefficient, *λ*. Based on a large amount of experimental data, empirical formulas were obtained to describe the relation of *λ* and corrosion potential of RC, and results proved that the CR obtained is more accurate when the value of *λ* is about 0.2–0.6 for activated steel, and 0.5–0.8 for passive steel. In addition, Xu et al. [132] investigated the error level of LPR caused by IR drop, in order to accurately evaluate corrosion status of CSB in chloride-contaminated concrete, and concluded that the error level of CSB in passivation state is relatively lower than that in active state.

LPR technology is widely used in experimental research and field testing because of its several merits, such as convenient instrument, real-time and online monitoring measurement, ability to evaluate corrosion of CSB in detail. The accuracy of LPR in laboratory tests, as the main electrochemical detection method, is comparable to that of GWL, regardless of concrete quality, cover thickness, and chloride concentration, as described in Equation (11) [133].

(11) $$i_{corr,GWL}=\;{0.86\cdot i}_{corr,LPR}$$

On the other hand, LPR is frequently applied for testing corrosion inhibitors, such as Faritov et al. [134]. However, there are some important considerations when adopting LPR in engineering applications: (1) To obtain the amount of corrosion, it needs the frequent LPR measurements. (2) Although the direct measurement of CR can be provided, it has a limitation in distinctly separate contributions of electrochemical parameters, including concentration polarization, charge transfer resistance, interfacial layers, and concrete resistance. (3) Based on steady state conditions, LPR can only provide information about general corrosion. Therefore, the method usually works with other techniques to ensure the results’ accuracy. (4) The medium with poor conductivity, especially when there is a dense layer of passivation (or oxide) film and corrosion products on the measurement device surface, introduces capacitance in the system and leads to an inaccurate LPR measurement. 

### 6.4. Tafel Extrapolation (TE)

**Figure 11:**
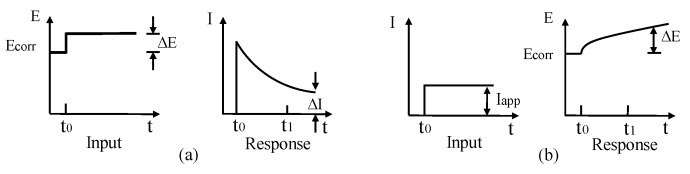
(**a**) Potentiostatic measurement; (**b**) galvanostatic measurement.

TE technique is an extension of LPR approach to determine CR based on corrosion current and Tafel slopes. Both LPR and TE techniques can either be achieved by applying constant current followed by potential (galvanostatic) measurement or applying constant potential followed by the current (potentiostatic) measurement, as shown in Figure 11.

For LPR technique, the variation in potential is limited within a small tolerance of ±25 mV, whereas for TE technique, the variation in potential can go beyond ±250 mV. Another difference is to explain the testing results for calculation CR. In TE, CR can be straightforwardly determined via a simple substitute Tafel slope values ($\beta_{a}$ and $\beta_{c}$) into Equation (12) to obtain corrosion current and then CR with Equation (13) [104].

(12) $${I\;=\;I}_{corr}\left\{\exp\left[\frac{{2.303(E\;-\;E}_{corr})}{\beta_{a}}\right]\;-\;\exp\left[\frac{{2.303(E\;-\;E}_{corr})}{\beta_{c}}\right]\right\}$$

(13) $$\mathrm{Corrosion}\;\mathrm{rate}\;\left(\mu m/\mathrm{yr}\right)=\frac{{0.129I}_{corr}\mathrm{EW}}{\mathrm{dA}}$$

where *I* and *E* are the current and potential at any time, respectively; *E_corr_* is the corrosion potential; A and *d* are surface area and density of CSB, respectively. EW is the equivalent weight of steel.

Usually, the rapid determination of CR by TE technique has the advantage in such research as corrosion inhibitor evaluation (Hasanov [135]; El-Maksoud [136]) and alloy comparison (Amin M A [137]).

### 6.5. Galvanostatic Pulse Transient Method (GPT)

**Figure 12:**
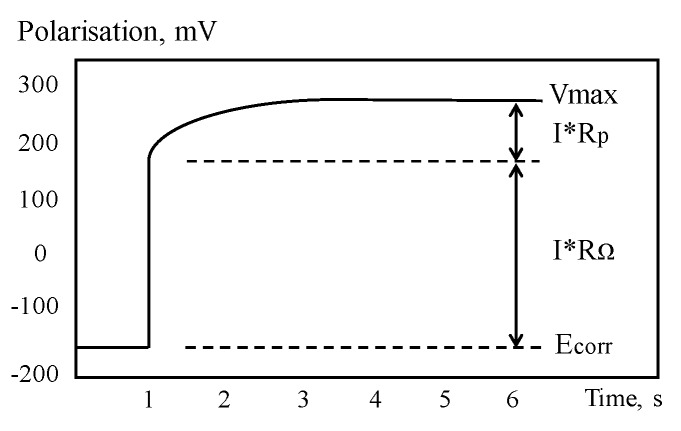
Typical potential–time curve.

GPT is a powerful tool for assessing CR of CSB through measurement of polarization resistance and has been progressively developed since 1988s [138]. For GPT, a small constant-current perturbation, I_app_, is performed to CSB in a way similar to the galvanostatic LPR, which is normally in the range of 5~100 A, and typical pulse duration is varied in 5 and 30 s. After CSB is polarized, the attenuation curve of potential in Figure 12 can be recorded by measuring the change in electrochemical potential between the reference electrode and CSB. The curve is analyzed to obtain the instantaneous quantitative information of corrosion condition.

Based on Randles circuit as the equivalent circuit of RC corrosion system at time of polarization by constant current, the transient behavior of CSB potential, *Vt*, can be described as Equation (14). However, Randles model, composed of a resistor in parallel with a single capacitor, is too simple to describe the corrosion interface of CSB and concrete. The experimental results by So et al. [139] proved that a model of a series of parallel components, including resistors and capacitors, can better simulate the corroded system. Birbilis et al. [140] proposed an analysis method that allows for more accurate measurement of certain electrochemical parameters, which is based on the non-exponential model using modified Kohlrausch Williams Watt formalism.

The values of *R_p_*, *C_dl_*, and *R*_Ω_ can be determined by optimizing the fitting of potential curve with experimental data. Though the linearization method, Equation (14) can be transformed into a linear form using Equation (15).

(14) $$V_{t}=I_{app}R_{\Omega }+I_{app}R_{\Omega }\left[1-\exp\left(-t/R_{p}C_{dl}\right)\right]$$

(15) $$\mathrm{In}\left(V_{max}-V_{t}\right)=In\left(I_{app}R_{\Omega }\right)-t/\left(R_{p}C_{dl}\right)$$

where *V_max_* is the steady state potential value after long polarization. After that, corrosion current *I_corr_* (A/cm^2^) can be obtained according to the Stern–Geary equation using Equation (10).

**Figure 13:**
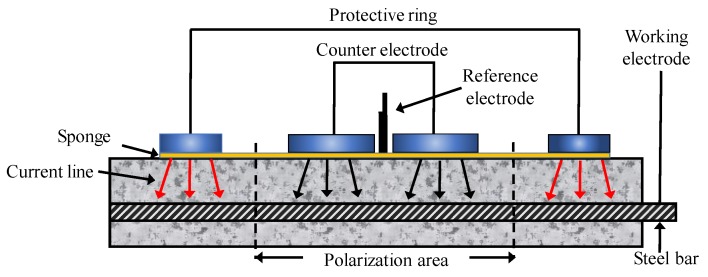
Polarization area of reinforcing steel.

When using GPT technique for long-term monitoring, it is essential to pay attention to the temperature and humidity in the environment during the test. In addition, the test specimens should be fully wetted to ensure good measurement accuracy, and the pulse current should be reduced as much as possible to improve detection precision and measurement stability. A guard ring can be used to limit electrical signals emitted from counter electrodes, and to determine the area of CSB affected by electrodes. The induced current makes bars in the guard ring polarized, and the applied current is limited in guard ring, thus, the affected area is as illustrated in Figure 13.

GPT technique has an advantage in the precise determination of active/passive corrosion of CSB under optimal conditions in which, generally, the pulse duration and the anodic pulse are set at 10 s and 50 μA, respectively. CR can be quickly determined for mapping the electrochemical characteristics of CSB, including double layer capacitance, half-cell potential, time constant *k*, *R_P_*, and *i_corr_*. Furthermore, GPT offers numerous advantages, such as its rapidity in a few seconds per measurement, high precision, and immunity to environment factors and thickness of concrete protection layer. Considering the aforementioned advantages, GPT is a promising choice for both laboratory and field measurement, especially in following conditions: (1) Thicker concrete layer. CR of CSB decreased with the increasing of concrete cover thickness [141]. (2) In the measurement of aging structure (dry concrete), the determination of CR using GPT method is in good agreement with that via GWL [142]. (3) Under the adverse conditions of the unknown information of CSB area or no requirement to the stable reference electrode, GPT technology would be a better choice if compared with HCP, LPR, and EIS [143].

From the reported literature, it is observed that works on the potential response of CSB to the different corroding environments, and the influencing degree of factors on GPT experimental results in on-site measurements, are relatively minimal; hence, further work will be needed.

### 6.6. Electrochemical Impedance Spectroscopy (EIS)

**Figure 14:**
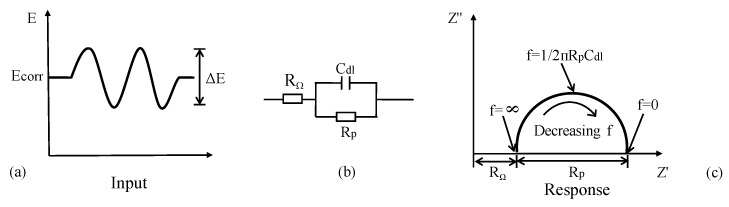
AC impedance of an electrochemical corrosion system: (**a**) applied disturbance voltage; (**b**) equivalent electrical circuit model; (**c**) nyquist plots.

As a transient spectrum analysis technique, EIS has wide application in assessing the kinetics of electrochemical reactions [144,145] and the mechanism of CSB corrosion protection than LPR measurement using DC perturbation. An AC voltage (10–20 mV) performed over a range of discrete frequencies (10^−2^–10^5^ Hz) is applied to disturb electrode in Figure 14a. Based on the relation between impedance and frequency, a more or less equivalent electrical circuit can describe the corrosion behavior of CSB [146]. For example, a corrosion interface typically comprises an interfacial polarization ohmic resistance *R_p_* and a double-layer capacitance *C_dl_* in parallel, as shown in Figure 14b. The impedance is given by

(16) $$Z\;=\;(\frac{1}{R_{p}{\;+\;j\omega C}_{dl}}{)}^{-1}$$

(1) In Nyquist, the plot of imaginary part against real part of impedance is basically a semicircle with a diameter of *R_p_*, whose center is offset from the origin by a value of *R_p_*/2 + *R_Ω_*. The double-layer capacitance *C_dl_* is obtained from frequency *f* in Figure 14c, which is located at the highest point on the semicircle using Equation (17):

(17) $$C_{dl}\;=\;\frac{1}{{2\pi R}_{p}f}$$

In practice, there will appear a combination of several different semicircles in the AC impedance response due to film effects, diffusion effects, etc. The radius of capacitance arc is closely related to CR. The charge transfer resistance becomes larger with a larger capacitance arc radius, however, CR of CSB will be smaller. *C_dl_* is useful for characterizing the response and identifying the part that is associated to corrosion processes. *R_p_* is normally used to obtain CR using Equations (3) and (10).

(2) In Bode, there are the logarithm of impedance (log|*Z*|) and the phase angle (*Φ*) on ordinate axis, and the logarithm of angular frequency (log*ω*, or logarithm of frequency, log*f*) on abscissa axis.

**Figure 15:**
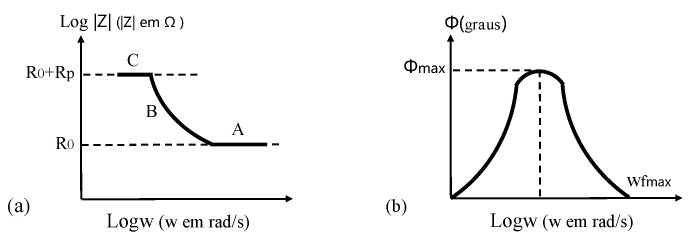
Bode plots of an electrochemical corrosion system: (**a**) the impedance modulus as a function of the angular frequency; (**b**) the phase angle as a function of the angular frequency.

In Figure 15, it is possible to determine *R_Ω_*, *R_p_*, and *C_dl_*, knowing that

(18) $$R_{p}\;=\;2\left|Z\right|\mathrm{tgmax}$$

(19) $$\omega_{fmax}=\frac{1}{C_{dl}R_{p}{{(1\;+\;R}_{p}{/R}_{\Omega })}^{1/2}}$$

where *f_max_* is the maximum phase angle of system impedance, and *ω_fmax_* is the angular frequency corresponding to *f_max_*. Three clear regions with different frequency are shown in Figure 15a, where A is characterized by passivation film or additional coating on CSB, B represents changes in electrical conductivity of coating during corrosion procedure, and *C* is related to corrosion reaction on metal/coating interface.

Generally, EIS method is the highly reproducible and powerful method for studying RC systems involved in CSB corrosion mechanism [147], corrosion factors, corrosion evolution process [148], and effectiveness validation of concrete repair scheme [149,150]. For a steel/concrete system, the important parameters including concrete characteristics, interfacial corrosion, surface films, and transport phenomenon, can be provided. In particular, EIS has made great progress on corrosion process of material coated Fe–C alloy film whose composition is similar to CSB [95]. In addition, the main advantages of EIS are (1) The electrochemical corrosion mechanism occurs by activation, diffusion, or concentration, and evolution of active/passive state over time can be identified; (2) The state and morphology of CSB corrosion will be characterized; (3) The performed signals with small amplitude can change the potential of corrosion. 

However, EIS has limitations in practical engineering application, because of the following unaddressed issues: (1) EIS is more suitable for laboratory researches, as its results analysis becomes more difficult by establishment of equivalent circuits that vary with corroded environment [151] and concrete heterogeneity. (2) Measurement of low CR requires low frequency AC signals, which make measurement difficult. (3) Geometric size of components has effect on impedance spectrum. In order to carry out field measurement, the spatial distribution of impedance must be considered. (4) There are no internationally accepted standards.

Recently, many researchers have focused on the comparison of different electrochemical methods. The comparative study on three main electrochemical methods (LPR, GPT, and EIS) by Shi et al. [152] suggest that all of them are capable of providing a reliable assessment for the corrosion state of CSB with good correlation. In addition, Vedalakshmi et al. [142] obtained the comparative conclusion that the lowest CR, of even less than 1 μm/year, can be predicted by only EIS.

### 6.7. Harmonic Analysis (HA)

HA technology has been strongly employed for corrosion in recent years, which is associated with the impedance method and provides faster execution and more direct results than those of EIS [153]. In the HA method, polyharmonic signals are produced in response to the perturbation, based on exploiting the non-linear properties of electrochemical corrosion process. By performing an AC voltage perturbation *V*_0_ (10 mV) at a single frequency in the same manner as EIS, measurement for AC current density *i*_1_, as well as for two higher harmonics, *i*_2_ and *i*_3_, can be achieved. Corrosion interface, played as a rectifier, is responsible for the creation of harmonics, and the response of second-harmonic current is a non-linear function of free corrosion potential.

This technique offers an advantage of easy assessment, in which both Tafel coefficient and corrosion current can be acquired by one measurement using Equations (20) and (21) [154]. B can then be calculated by Tafel constants obtained using Equation (22) [155].

(20) $$i_{corr}\;=\;\frac{i_{1}^{2}}{\sqrt{48}\sqrt{{2i}_{1}i_{3}{-i}_{2}^{2}}}$$

(21) $${1/\beta }_{a}{\;\mathrm{or}\;1/\beta }_{c}{\;=\;1/2V}_{0}{(i}_{1}{/i}_{corr}{\;+\;4i}_{2}{/i}_{1})$$

(22) $${1/\beta }_{a}{\;\mathrm{or}\;1/\beta }_{c}{\;=\;1/2V}_{0}{(i}_{1}{/i}_{corr}{\;+\;4i}_{2}{/i}_{1})$$

On the other hand, HA technique has the specific advantage of measuring corrosion current directly, according to the harmonic currents, and does not need Tafel constants for a reliable determination of CR, which is an essential parameter to other electrochemical methods, such as LPR, EIS, and TE [156,157]. Furthermore, Lawson et al. [158] obtained a frequency and amplitude values of 100–150 MHz and 10 mV, respectively, dependency in HA, which gives a reliable CR of CSB in concrete, consistent with GWL. 

Nevertheless, HA, like EIS and LPR, makes its calculation of penetration rates under the assumption of uniform corrosion. The measurement is most unreliable if localized corrosion damage happens, and data is only suitable for qualitative analysis. Therefore, the HA technique is worthy of further study for wide applications.

### 6.8. Electrochemical Noise (EN)

EN technology has made great progress in the field of corrosion and protection science. EN is referred to a kind of fluctuation caused by a change in potential and current of two electrodes, along with the process of passivation or corrosion on CSB surface. Frequency is typically in range of 10^−3^ to 1.0 Hz, and the voltage is in the order of μV to mV, whereas the current is in the order of nA to μA. The more intense the damage and repair process is, the higher the frequency of noise potential and current fluctuation. Analysis of the obtained noise spectra not only indicates corrosion process, but also determines corrosion characteristics. It is generally believed that the noise resistance is inversely proportional to CCD. The *R_n_* is given by

(23) $$R_{n}{\;=\;\sigma }_{E}{/\sigma }_{I}$$

(24) $$i_{corr}{\;=\;B/R}_{n}{\;=\;B\sigma }_{I}{/\sigma }_{E}$$

where *σ_E_* and *σ_I_* are the standard deviation of potential and current, respectively; *B* is the coefficient of Stern–Geary. The noise signal can be presented in form of power spectra in frequency domain, generated from the signal in time domain using MEM (maximum entropy method) or FFT (fast Fourier transform) in spectral analysis. However, for the metastable system and unstable passivation system, chaos theory is the recommended method. 

By combining other technologies [159], EN technology is useful tools for monitoring corrosion status and identification of corrosion types, such as uniform corrosion [160], especially for pitting corrosion [161,162] and galvanic corrosion [163]. Furthermore, EN is used to investigate the inhibition performance of novel inhibitors for steel, like LPR and EIS [164]. On the other hand, Nagiub et al. [165] used EN and EIS techniques to investigate corrosion behavior of steel to obtain the connection between *R_p_* and noise resistance Rn, in which Rn was generally much smaller than *R_p_*. In some media of low ionic conductivity, such as crude oil, where it is difficult to use electrochemical techniques due to continuous current experiments, EIS and EN have been proposed as viable methods [166]. 

EN technique is a straightforward NDT, however, specific issues, such as the noise from instrument, aliasing, and quantization error, have to be taken into consideration. Therefore, there is a pressing need for developing more advanced analytical methods in current EN technology and exploring wider application areas. Similar to the research by Hardon et al. [167], a reproducible correlation between standard deviation of potential noise and CR was obtained to provide an important basis.

## 7. Three Electrochemical Factors (TEF)

Usually, a corrosion evaluation method based on a single factor as the criterion is restricted to making an accurate assessment CR of CSB, as it is subject to influence by various factors and uncertainties in the concrete environment. Hence, TEF combining three factors, including corrosion potential, corrosion current, and concrete resistivity, is introduced to assess corrosion severity of CSB, which ameliorates the veracity of some standards, such as ASTMC 876-91.

Based on a large amount data collected by several techniques, TEF evaluation system realizes the effective use of discriminant analysis under Fisher criterion to quantitatively evaluate corrosion extent. TEF factors are used as variable element to establish a three-element discriminant function. The reliability of corrosion classification is checked by calculating the posterior probabilities of new test pieces using Bayes statistics. Yang et al. [168] verified the feasibility and reliability of TEF method for evaluating corrosion of CSB by experiments. 

Although TEF technology has not been widely used in practical engineering, it serves as a good example of comprehensive application combining various detection methods, and standardization of corrosion assessment. Further work will be needed, such as the incorporation of other electrochemical factors into the discriminant function, to improve its accuracy and reliability.

## 8. Conclusions

CSB corrosion has been considered as a major issue causing widespread premature deterioration of RC structures, in particular, to aging structures and marine constructions, and even bringing economic burden and safety risks. Hence, corrosion monitoring techniques play an effective and significant role in assessing the structural health and ensuring their lifecycle. This paper carries out a systematic classification of these diagnostic methods, including visual inspection, empirical analysis, physical methods, and electrochemical methods, and reviews their advantages and limitations in order to give a reference point for their better development in the future, and widen the range of application. Moreover, the three electrochemical factors method is introduced to offer a desirable way to combine various techniques to improve the corrosion evaluation accuracy. 

To help assess corrosion status and select the suitable corrosion diagnostic technique, a tabulated comparison of the types of technologies is shown in Table 2, according to their respective characteristics and application limitations. In the present status, the corrosion detections need further works in the following parts: (1) although, numerous capabilities, such as convenience, efficiency, and accuracy, help NDT technologies as a strong candidate for detecting occurrence of corrosion in real-time. However, GWL measurement, as the most reliable reference method, is fundamental to validate the accuracy of other NDT methods, and even certain corrosion prevention measures. (2) Based on a comprehensive consideration of diagnostic techniques, preliminary methods, such as ECT, AE, IT, and radiography, can be considered in the early stage of corrosion, and the ensuing investigation can be done using other methods, like HCP, EIS, LPR, etc., to attain a more extensive identification of corrosion. (3) The packaging and embedding technology of FOCS in concrete needs further research, in order to avoid different attacks and ensure longevity throughout the whole lifetime. Although FOCS is widely used in experiments, there is a limitation on its fragility and service life on glass fiber; hence, the polymer optical fiber needs further work regarding applications in the field of corrosion. (4) There is room for developing novel sensors, with more accuracy, intelligence, convenience, durability, etc. One of the most important parameters of sensors is their service life in the whole lifecycle of RC structures, as the replacement of a new sensor could cause certain disturbance or damage to structures. (5) As indicated previously, the precision of CSB corrosion status, evaluated by a single monitoring index, is unfavorable in experimental and situ measurement. TEF method exhibit the immense potential of designing comprehensive corrosion assessment based on various factors, in order to acquire information about the corrosion state from different perspectives.

## Figures and Tables

**Table 1:** Typical prediction models of CSB corrosion.

Model Classification		Characteristics
Empirical model [14,15]		1) Their model-building by regression analysis based on experimental and field data; 2) Easy operation, but limited application.
Reaction-control model	Oxygen diffusion [16,17]	1) Its theoretical principle mainly considers two important factors controlling corrosion reaction; 2) The corrosion analysis without considering of electrochemistry principles; 3) The application of important parameters affecting corrosion propagation in model-building process, such as external loads and cracks [22,23].
	Resistivity [18,19]	
	Coupling of oxygen and resistivity [20,21]	
Electrochemical model	Based on Butler–Volmer dynamics [24,25]	1) Based on corrosion reaction dynamics, the relationship between current density of two electrodes and other electrochemical parameters is well established; 2) The requirement of complex corrosion electrochemistry theory makes it difficult to popularize in practical engineering.
	Model based on other theories [26]	

**Table 2:** Technologies’ performance and comparison criteria.

**Methods**	**Visual Inspection**	**Empirical Analysis**		**ERP**	**ECT**	**AE**
Non-perturbing	No	No		Yes	Yes	Yes
Sensitivity	Low	Low		Medium	Medium	Medium
Measurement speed	Slow	Slow		Fast	Medium	Medium
Applicability	Both (field and experiment)	Experiment		Both	Experiment	Both
Obtained information	Qualitative	Qualitative (based on empirical data)		Quantitative	Semi-quantitative	Qualitative
Measurement parameter or data type	Average CR	Predicted CR		Mass loss of CSB	Corrosion probability	Corrosion probability
Types of corrosion assessed	General and local corrosion	General corrosion		General corrosion	General and local corrosion	Early stages of corrosion and different crack types
Data interpretation	Simple, but superficial	Simple, but inaccurate		Simple and accurate	Visual and accurate	Relatively inaccurate
Advanced instruments or special requirements	Convenient instrument and experience needed	Convenient instrument and experience needed		Convenient instrument, easy and safe operation	Convenient instrument, easy and safe operation	Convenient instrument, easy and safe operation
**Methods**	**Radiography**	**IT**		**FOCS**	**HCP**	**CRM**
Non-perturbing	Yes	Yes		Yes	Yes	Yes
Sensitivity	Low	Medium		High	Medium	Medium
Measurement speed	Slow	Medium		Fast	Fast	Fast
Applicability	Experiment	Experiment		Both	Both	Both
Obtained information	Qualitative	Qualitative		Quantitative	Qualitative	Qualitative
Measurement parameter or data type	Images and ray value	Corrosion probability		CR	Corrosion probability	Corrosion probability
Types of corrosion assessed	General and local corrosion	General and local corrosion		General corrosion	General corrosion	General corrosion
Difficulty level of data interpretation	Visual, but superficial	Visual and intuitive		Simple and accurate	Simple and intuitive	Simple, but relative inaccurate
Advanced instruments or special requirements	Bulky and costly equipment, highly technical and hazardous	Convenient instrument, easy and safe operation		Convenient instrument, easy and safe operation	Convenient instrument, easy and safe operation	Convenient instrument, easy and safe operation
**Methods**	**LPR**	**TE**	**GPT**	**EIS**	**HA**	**EN**
Non-perturbing	No	No	No	No	No	Yes
Sensitivity	High	High	High	High	High	High
Measurement speed	Fast	Fast	Fast	Medium	Medium	Medium
Applicability	Both	Experiment	Both	Experiment	Experiment	Experiment
Obtained information	Quantitative	Quantitative	Quantitative	Quantitative	Quantitative	Quantitative
Measurement parameter or data type	CR	CR	CR	CR and corrosion mechanism	CR	CR
Types of corrosion assessed	General and galvanic corrosion.	General and local corrosion	Active/passive corrosion	General corrosion	General corrosion	General, local and pitting corrosion
Difficulty level of data interpretation	Relatively difficult (IR drop)	Simple and accurate	Simple and accurate	Relatively difficult (Equivalent circuits)	Simple and accurate	Difficult (sophisticated mathematics)
Advanced instruments or special requirements	Convenient instrument, easy and safe operation	Convenient instrument, easy and safe operation	Convenient instrument, easy and safe operation	Costly equipment, easy and safe operation	Convenient instrument, easy and safe operation	Convenient instrument, easy and safe operation

## Abbreviation

CSB	steel bar embedded in concrete	CSE	copper sulfate electrode
RC	reinforced concrete	DC	direct current
CCD (*i_corr_*)	corrosion current density	AC	alternating current
CR (ν)	corrosion rate	ρ	concrete resistivity
I_corr_	corrosion current	*R_p_*	polarization resistance
*t*	corroding time	*C_dl_*	double layer capacitance of RC interface
S	corroded surface area of CSB	*R_Ω_*	concrete resistance between reference electrode and CSB
D	diameter of CSB	$\beta_{a}$	Tafel constants of anode
L	length of CSB	$\beta_{c}$	Tafel constants of cathode
$m_{i}$	initial quality before corrosion	*B*	Stern–Geary constant
$m_{f}$	quality after corrosion	λ	compensation coefficient
*ϱ*	degree of corrosion	*E_corr_*	corrosion potential
$\omega_{m}$	molar mass of Fe	*E*	potential at any time
V	electrons transferred number	*I*	the current at any time
F	Faraday constant	*d*	density of CSB
*CS*_0_	initial cross-section	*I_app_*	applied constant-current perturbation
*R*_0_	initial electrical resistance	Z	impedance in EIS
ΔR	change in electrical resistance	*i*_1_	first harmonic currents
CF	initial circumference of ERPs	*i*_2_	second harmonics currents
RCS	cross-section reduction of CSB	*i*_3_	third harmonics currents
$T_{c}$	corroded layer thickness of CSB	*R_n_*	noise resistance
SHM	structure health monitoring	*Φ*	phase angle
RI	reflective index	*ω*	angular frequency
SCE	saturated calomel electrode	*f*	frequency
